# The mucin-like, secretory type-I transmembrane glycoprotein GP900 in the apicomplexan *Cryptosporidium parvum* is cleaved in the secretory pathway and likely plays a lubrication role

**DOI:** 10.1186/s13071-022-05286-8

**Published:** 2022-05-17

**Authors:** Xiaohui Li, Jigang Yin, Dongqiang Wang, Xin Gao, Ying Zhang, Mingbo Wu, Guan Zhu

**Affiliations:** Key Laboratory of Zoonosis Research of the Ministry of Education, The Institute of Zoonosis, The College of Veterinary Medicine, Jilin University, Changchun, 130062 China

**Keywords:** Apicomplexan, *Cryptosporidium parvum*, Microneme proteins, Glycoprotein GP900, Domain cleavage

## Abstract

**Background:**

*Cryptosporidium parvum* is a zoonotic parasite and member of the phylum Apicomplexa with unique secretory organelles, including a rhoptry, micronemes and dense granules that discharge their contents during parasite invasion. The mucin-like glycoprotein GP900 with a single transmembrane domain is an immunodominant antigen and micronemal protein. It is relocated to the surface of excysted sporozoites and shed to form trails by sporozoites exhibiting gliding motility (gliding sporozoites). However, the biological process underlying its relocation and shedding remains unclear. The primary aim of this study was to determine whether GP900 is present as a transmembrane protein anchored to the plasma membrane on the surface of sporozoites and whether it is cleaved before being shed from the sporozoites.

**Methods:**

Two anti-GP900 antibodies, a mouse monoclonal antibody (mAb) to the long N-terminal domain (GP900-N) and a rabbit polyclonal antibody (pAb) to the short C-terminal domain (GP900-C), were produced for the detection of intact and cleaved GP900 proteins in sporozoites and other parasite developmental stages by microscopic immunofluorescence assay and in discharged molecules by enzyme-linked immunosorbent assay.

**Results:**

Both anti-GP900 antibodies recognized the apical region of unexcysted and excysted sporozoites. However, anti-GP900-N (but not anti-GP900-C) also stained both the pellicles/surface of excysted sporozoites and the trails of gliding sporozoites. Both antibodies stained the intracellular meronts, both developing and developed, but not the macro- and microgamonts. Additionally, the epitope was recognized by anti-GP900-N (but not anti-GP900-C) and detected in the secretions of excysted sporozoites and intracellular parasites.

**Conclusions:**

GP900 is present in sporozoites and intracellular meronts, but absent in sexual stages. It is stored in the micronemes of sporozoites, but enters the secretory pathway during excystation and invasion. The short cytoplasmic domain of GP900 is cleaved in the secretory pathway before it reaches the extracellular space. The molecular features and behavior of GP900 imply that it plays mainly a lubrication role.

**Graphical Abstract:**

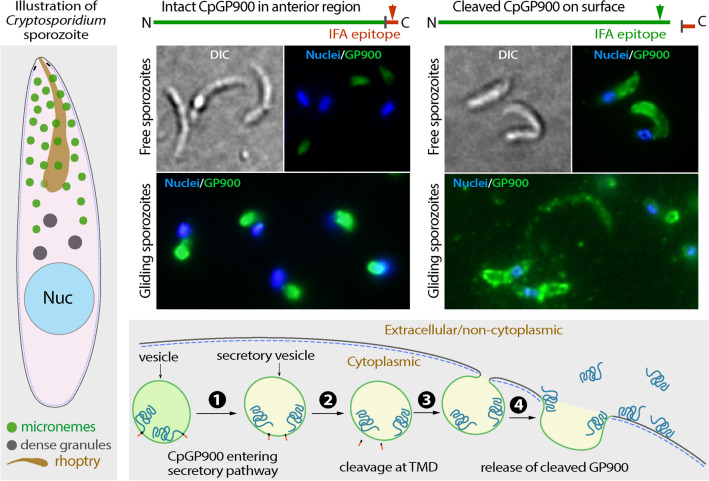

## Background

*Cryptosporidium parvum* is a zoonotic apicomplexan parasite that infects humans and a wide range of animal species. It is transmitted between human and animal hosts by the oral–fecal route via the shedding of oocysts, an environmental stage of the parasite, in the feces of infected organisms and subsequent ingestion of these oocysts by other organisms. A round oocyst (approx. 5 μm) contains four banana-like sporozoites (approx. 5 × 1 μm) that are released in the gastrointestinal tract by an excystation process following ingestion of the oocyst by the host. Released sporozoites invade host enterocytes, coming to reside on top of the host cells in a parasitophorous vacuole surrounded by a host cell-derived membrane termed the parasitophorous vacuole membrane (PVM). Before the establishment of infection, sporozoites move through the intestinal mucus by active gliding locomotion to locate on the surface of host cells. At the infection site, a sporozoite attaches to the host cell, which forms a PVM engulfing the parasite; the sporozoite is then transformed from a banana-like shape to a small round trophozoite in the PVM. During the gliding and invasion processes, a number of proteins are discharged from several specialized secretory organelles called the micronemes, rhoptries and dense granules.

**Fig. 1:**
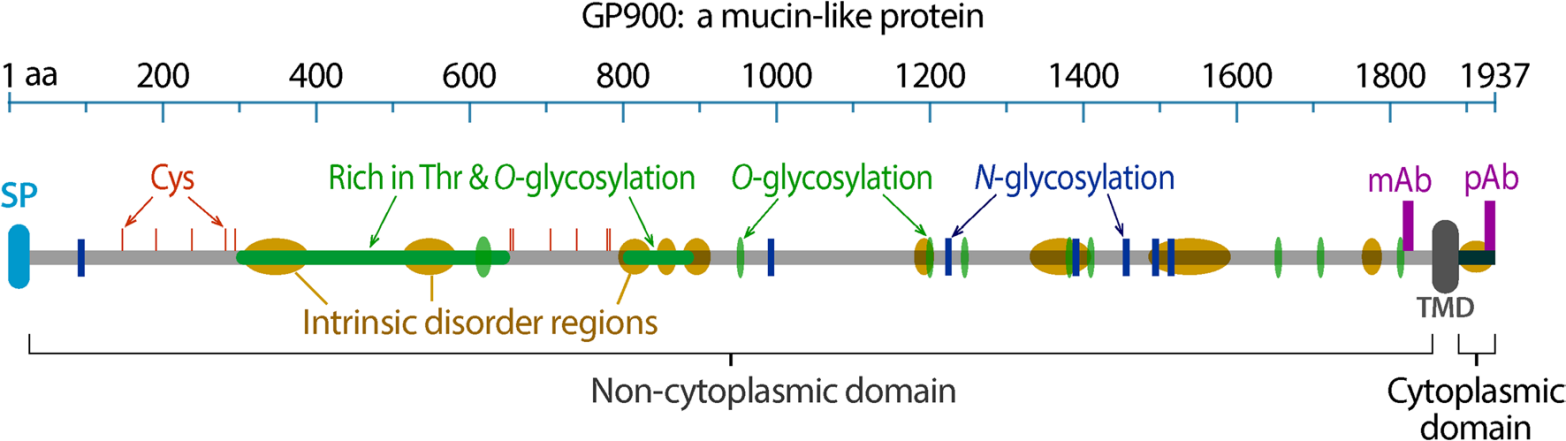
Architecture of the mucin-like *Cryptosporidium parvum* GP900 protein. The protein contains an N-terminal SP for targeting the protein to the endoplasmic reticulum and a TMD near the C-terminus to separate the long non-cytoplasmic domain and the short cytoplasmic domain. There are 10 intrinsic disorder regions with various lengths across the protein (brown), numerous sites for *O*-glycosylation, including two long threonine (Thr)-rich regions (green), a number of sites for *N*-glycosylation (blue), and 15 cysteine (Cys) residues (red). Sites recognized by the mouse anti-GP900-N monoclonal antibody (mAb) and the rabbit anti-GP900-polyclonal antibody (pAb) are also marked (purple). Abbreviations: SP, signal peptide; TMD, transmembrane domain

One of the molecules currently known to be secreted from the micronemes is a mucin-like glycoprotein called GP900 (or CpGP900). This glycoprotein is encoded at the locus cgd7_4020 based on the nomenclature for the genome of Iowa-II strain of *Cryptosporidium parvum*. It is defined by an intronless 5814-bp open reading frame as a 1937-amino acid (aa) protein that contains a single transmembrane domain (TMD) near the C-terminus. Based on its amino acid composition, the predicted molecular weight of GP900 is 202.6 kDa, whereas its native protein might migrate at a position close to or larger than 900 kDa due to its heavy *O*-linked glycosylation and numerous *N*-linked glycosylation (Fig. 1).

GP900 was first reported 30 years ago as a > 900-kDa glycoprotein and one of the antigens recognizable by hyperimmune bovine colostral immunoglobins [1] and a mouse antiserum affinity-purified with the clone S34 in a λgt11 *C. parvum* genomic expression library [2]. It was later defined as a micronemal protein in sporozoites in intact oocysts and merozoites contained in the PVM, but which relocated to the surface of excysted sporozoites capable of forming trails during gliding locomotion, mediating sporozoite invasion [3]. It was also suggested to be one of the molecules tethering the sporozoites to the inner side of the oocyst walls [4]. Mass spectrum-based evidence indicated for *O*-linked and *N*-linked glycosylation for GP900, both in relatively simple and unextended forms (also see illustration in Fig. 1) [5, 6].

Overall, the immunodominant antigen GP900 was implied to be involved in the adhesion and invasion of *Cryptosporidium* zoites, although the molecular process during the parasite invasion remained unknown. Here we provide experimental evidence showing that *C. parvum* GP900 (CpGP900) stored in the sporozoite micronemes is cleaved in the secretory pathway and discharged to the cell surface and extracellular space of excysted sporozoites in a form lacking the C-terminal cytoplasmic domain. Consequently, secreted CpGP900 is unable to anchor to the sporozoite surface due to its lack of a transmembrane domain, suggesting that this glycoprotein likely plays a lubrication role during interaction with host cells.

## Methods

### Parasite materials and general in vitro cultivation protocol

An isolate of *C. parvum* (subtype IIaA17G2R1 based on *gp60* gene) was propagated in calves in-house, and oocysts were purified by sucrose gradient centrifugation as previously described [7, 8]. Free sporozoites were prepared by an in vitro excystation protocol, in which oocysts were incubated in a serum-free RPMI-1640 medium containing 0.75% taurodeoxycholic acid at 37 °C for 45–60 min. In vitro culture of *C. parvum* was performed using the HCT-8 cell line (human ileocecal colorectal adenocarcinoma; ATCC # CCL-244) to host parasite growth. HCT-8 cells were cultured in RPMI-1640 medium supplemented with 10% fetal bovine serum, 2 mM l-glutamine, 50 units/ml penicillin and 50 μg/ml streptomycin at 37 °C in 5% CO_2_. For immunofluorescence microscopy assay (IFA), host cells were seeded onto 48- or 24-well plates containing poly-l-lysin-treated round glass coverslips and infected with *C. parvum* by inoculation with either free sporozoites or oocysts [9, 10]. The invasion stage was prepared by infecting host cell monolayers (60% confluence) with freshly excysted sporozoites for 15–30 min [9, 10]. Intracellular stages were prepared by inoculating host cell monolayers with oocysts (viability > 80% based on in vitro excystation rate) at 37 °C for 3 h, followed by the removal of uninvaded parasites. Invaded parasites were allowed to grow for various time periods, varying from 3 to 72 h post-infection (hpi). For the real-time quantitative PCR assay(qRT-PCR), host cells were cultured on 96-well plates containing no slides for specified times. All samples prepared as described were fixed with formaldehyde, lysed for preparing total RNA or processed as described in following sections.

### Antibody production

Two antibodies against CpGP900 were produced in this study: (I) a rabbit polyclonal antibody (pAb) against a short peptide located on the C-terminal cytoplasmic side of the TMD (^1923^TVVTIERDSSFWNES^1937^, named GP900-C), and (ii) a mouse monoclonal antibody (mAb) against a peptide on the N-terminal non-cytoplasmic side of the TMD (^1814^VPGTAAPKKGG^1825^, named GP900-N). Both peptides were synthesized by the China Peptides Company (Shanghai, China) and conjugated to keyhole limpet hemocyanin (KLH) as described [11]. For pAb production, two rabbits were immunized with the KLH-linked GP900-C peptide by subcutaneous injections using a standard 4-injection immunization protocol as previously described [9, 10, 12]. Serum samples were collected prior to the first injection and 14 days after the last one. Rabbit antiserums were subjected to affinity purification by nitrocellulose membrane blotting with peptide GP900-C as previously described [9, 10, 13]. For mAb production, two Balb/c mice were immunized four times with immunogen (KLH-linked GP900-N; 100 μg with Freund’s complete adjuvant for the first injection and 50 μg with incomplete adjuvant for next three injections) and a final intravenous injection of 20 μg immunogen containing no adjuvant. Standard protocols were used for hybridoma production [14, 15]. A clone GP900 AB5 was selected after three rounds of screening by enzyme-linked immunosorbent assay (ELISA) and used for producing mouse ascites.

### Western blot analysis

Parasite oocysts were disrupted by five freeze–thaw (liquid nitrogen–ice) cycles and lysed with a radioimmunoprecipitation assay (RIPA) buffer containing 1% Triton X-100 and a protease inhibitor cocktail for eukaryotes (Sigma–Aldrich, St. Louis, MO, USA) on ice overnight, followed by additional 1-h incubation at room temperature. Lysates equivalent to 1.0 × 10^7^ sporozoites/lane were heated with reducing sample buffer for 5 min at 95 °C, fractionated by 6% sodium dodecyl sulfate-polyacrylamide gel electrophoresis and transferred onto nitrocellulose membranes. The blots were blocked in 5% skim milk-TBST and probed with affinity-purified anti-GP900-C pAb (1:100 dilution in blocking buffer) or anti-GP900-N mAb (1:50 dilution in blocking buffer) for 1 h. After three washes with blocking buffer, the blots were incubated with horseradish peroxidase-conjugated secondary antibodies (i.e. goat anti-rabbit immunoglobulin G [IgG] or goat anti-mouse IgG; Invitrogen, Thermo Fisher Scientific, Waltham, MA, USA), and visualized using an enhanced chemiluminescence reagent (Beyotime Biotechnology, Shanghai, China) according to the manufacturer’s instructions. All procedures were conducted at room temperature unless specified otherwise.

### Immunofluorescence microscopy assay

Intact oocysts after disruption by the freeze–thaw cycles and excysted sporozoites of *C. parvum* were fixed in 4% formaldehyde for 30 min, washed with phosphate buffered saline (PBS) and applied to glass slides coated with poly-l-lysine. After air-drying for 1–2 h, samples were permeabilized with 0.2% Triton X-100 for 5 min and placed in a blocking solution containing 3% bovine serum albumin (BSA) in PBS for 1 h. In some experiments, the permeabilization step was omitted to enable antibody-labeling of proteins on the outside of the fixed sporozoites. In the gliding trail experiments, excysted sporozoites in RPMI-1640 medium were placed onto poly-l-lysine coated slides for 20 min at 37 °C, followed by the same fixation and permeabilization procedures as described. Invading sporozoites and intracellular meronts on host cell monolayers were prepared as described in “Parasite materials and general in vitro cultivation protocol” section, followed by fixation, permeabilization and blocking as described above.

For immunolabeling, samples were incubated with affinity-purified anti-GP900-C pAb or anti-GP900-N mAb in blocking buffer for 1 h, followed by three washes in blocking buffer and incubation for 1 h with one of the following secondary antibodies: Alexa Fluor 488-labeled goat anti-rabbit-IgG antibody, Alexa Fluor 488-labeled goat anti-mouse-IgG antibody (Thermo Fisher Scientific; 1:2000 dilution in PBS). In the dual-labeling assay of sporozoites, anti-GP900-C and anti-GP900-N antibodies were added together, and secondary antibodies used goat anti-rabbit and anti-mouse antibodies conjugated with Alexa Fluor 488 and Alexa Fluor 594, respectively. Some specimens were also co-stained with rhodamine-phalloidin to visualize host cell F-actin. Samples were then stained for nuclei with 4,6-diamidino-2-phenylindole (DAPI) (1.0 μg/ml). There were three washes with PBS between steps (5 min each). All procedures were performed at room temperature. Slides were examined under an Olympus BX53 research fluorescence microscope, and images were captured with an Olympus DP72 camera (Olympus Corp., Tokyo, Japan) and stored in TIFF format. The signal levels of images were linearly adjusted with Adobe Photoshop 2021 without local manipulations and presented with Adobe Illustrator 2021 (Adobe Inc., San Jose, CA, USA).

### qRT-PCR analysis of GP900 gene transcripts

Total RNA samples were isolated from oocysts, excysted sporozoites and intracellular parasites cultured in HCT-8 cells at 3, 6, 12, 24, 48 and 72 hpi using an iScript qRT-PCR sample preparation reagent (Bio-Rad Laboratories Inc., Hercules, CA, USA). The relative transcript level of *CpGP900* was detected by a SYBR Green-based qRT-PCR assay using HiScript II One-Step qRT-PCR SYBR Green Kit (Vazyme Biotech Co., Nanjing, China) with the primers 5′-ATC TAT TCC TCC AAG CGT ACC A-3′ and 5′-CAT TAT TGG GTT CAA GTC ACC A-3′. For quality control, the transcripts of the previously characterized lactate dehydrogenase (*CpLDH*) and elongation factor 1α (*CpEF1a*) genes were also detected using the primers as described previously [8, 16, 17]. The transcript of *C. parvum 18S* rRNA (*Cp18S*) was detected for normalization as previously described [8, 18].

The qRT-PCR assays were performed in a 20-µl reaction volume containing 0.2 µM of each primer, 1.0 µl One Step SYBR enzyme mix, 10 µl SYBR Green mix and 0.4 µl ROX reference dye 1 (50×) and 0.2 ng of total RNA isolated from oocysts/sporozoites or 15 ng total RNA from intracellular parasites. The cycling reactions were: 50 °C for 3 min to synthesize cDNA, 95 °C for 30 s to inactivate reverse transcriptase and 40 cycles at 95 °C for 10 s and 60 °C for 30 s to produce amplicons. Each sample included ≥ 2 biological replicates and ≥ 2 technical replicates in the qRT-PCR reactions. Relative transcript levels were calculated using an empirical 2^−∆∆CT^ formula as previously described [8, 18, 19].

### ELISA detection for the secretion of CpGP900

The secretion of CpGP900 proteins was evaluated by ELISA in excysted sporozoites, during invasion and in intracellular developmental stages between 3 and 6 hpi, 10 and 12 hpi and 20 and 24 hpi. Free sporozoites were prepared by in vitro excystation as described above. A portion of the sporozoites (1 × 10^7^) immediately after excystation was washed three times in PBS by centrifugation and resuspended in serum-free RPMI-1640 medium. After incubation at 37 °C for 1 h, supernatants were collected by centrifugation (12,000 *g*, 5 min). The remaining sporozoites were used to infect HCT-8 monolayers at approximately  90% confluence in 48-well plates (5 × 10^5^/well) for collecting the following samples. The invasion stage was prepared by inoculating excysted sporozoites (after three washes following excystation) in serum-free RPMI-1640 medium for 3 h, followed by the collection of supernatants (i.e. 0–3 hpi stage). Other intracellular stages were prepared by culturing parasites in serum-containing complete medium that was replaced by serum-free medium for specified time periods (i.e. 3–6 hpi, 10–12 hpi and 20–24 hpi). Supernatants were collected from the wells at the end of each time period, followed by replacement with complete medium and continuous culture to the next time period. The positive specimen control consisted of crude extracts prepared from excysted sporozoites (1 × 10^7^) by three freeze–thaw cycles and the collection of supernatants by centrifugation (12,000 *g*, 5 min).

To detect secreted CpGP900, supernatants prepared as described above were mixed with ELISA coating buffer (0.05 M carbonate-bicarbonate, pH 9.6) and added to 96-well ELISA plates (50 μl/well), followed by incubation overnight at 4 °C. The plates were then rinsed with PBS and blocked with 3% BSA in PBS (100 μl/well) at 37 °C for 1 h. After three washes with 0.05% Tween-20 in PBS (PBS-T buffer), the plates were incubated with affinity-purified pAb to GP900-C (1:5 dilution) or mAb to GP900-N (1:5 dilution) in PBS-T buffer (50 μl/well) at 37 °C for 1 h. A mouse antiserum (1:10 dilution) raised against total proteins in *C. parvum* sporozoites was used as the positive control for secreted proteins. After three washes in PBS-T buffer, the plates were incubated with alkaline phosphatase-conjugated goat anti-rabbit IgG (ImmunoWay Biotechnology Co., Plano, TX, USA; 1:10,000 dilution) or goat anti-mouse IgG(H+L) (ImmunoWay Biotechnology Co.; 1:10,000 dilution) secondary antibodies, followed by color development with *p*-nitrophenyl phosphate (Sigma-Aldrich) in accordance with the manufacturers’ instructions. Optical density at 405 nm (OD_405_) was read in a multifunctional microplate reader (BioTek, Winooski, VT, USA). Three independent experiments were performed, and each experiment included at least two biological replicates and two technical replicates.

## Results

### Molecular and domain features of the single-pass type I membrane GP900

The glycoprotein GP900 (1937 aa) is a single-pass membrane protein consisting of an N-terminal signal peptide (SP; positions 1–26 aa) and a single TMD (1863–1887 aa) near the C-terminus (Fig. 1). The presence of an SP and a TMD indicates that GP900 is a type I membrane protein in the secretory pathway, whereby GP900 is translated on the endoplasmic reticulum (ER) with the N-terminal domain inserted into the ER lumen for initial glycosylation and further glycosylation occurring in the Golgi body. The SP is cleaved in the ER after the completion of translation. It is predicted that the long N-terminal domain (approx. 1837 aa) and very short C-terminal domain (approx. 50 aa) are non-cytoplasmic and cytoplasmic, respectively. GP900 as a secretory glycoprotein was also confirmed in earlier studies, based on its presence in the secretory organelle micronemes in sporozoites and merozoites by IFA and immunoelectron microscopy (IEM) [1–3], and in the present study (described in following sections).

GP900 contains a large number of threonine (Thr; *n* = 503; 26%) and serine (Ser; *n* = 155; 8%) residues, including two large Thr-rich stretches that are heavily *O*-glycosylated, and an average number of asparagine (Asn) residues (*n* = 89; 4.6%) that are partially *N*-glycosylated (Fig. 1, marked in green or blue) [5, 6]. GP900 is an intrinsic disorder protein and possesses 10 intrinsic disorder regions across the entire protein (Fig. 1, marked in brown), suggesting its overall structure flexibility. GP900 also possesses 11 cysteine (Cys) residues (Fig. 1, marked in red), all located on the N-terminal half of the protein, suggesting some intramolecular (and potentially intermolecular) cross-links by disulfide bonds to increase the stability of the N-terminal half of the protein.

**Fig. 2:**
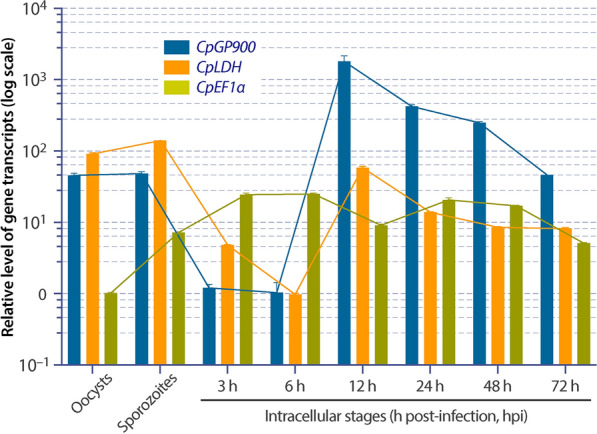
Relative levels of transcripts of the *C. parvum* GP900 (*CpGP900*), lactate dehydrogenase (*CpLDH*) and elongation factor 1α (*CpEF1α*) genes in oocysts, excysted sporozoites and intracellular parasites developed for various lengths of time as determined by qRT-PCR. The relative levels were normalized with those of the *Cp18S* transcript. *CpLDH* and *CpEF1α* were included in the assays and detected in parallel for comparison and quality control. Abbreviations: qRT-PCR, quantitative reverse transcription-real time PCR

The transcript of the *GP900* gene could be detected in oocysts, free sporozoites and intracellular developmental stages (Fig. 2). The transcript levels were relatively high in oocysts and sporozoites when normalized with the* Cp18S* transcript, but varied dramatically in the intracellular stages. The lowest levels were observed shortly after invasion (i.e. 3–6 hpi; representing early development of meronts), increased to the highest levels at 12 hpi (representing the maturation and formation of merozoites in the first generation of merogony) and then steadily declined from 24 to 48 hpi and at 72 hpi (representing the much less synchronized second and third generations of merogony and gametogenesis).

### The C-terminal cytoplasmic domain of GP900 is cleaved in the secretory pathway before reaching the cell surface in *C. parvum* sporozoites

**Fig. 3:**
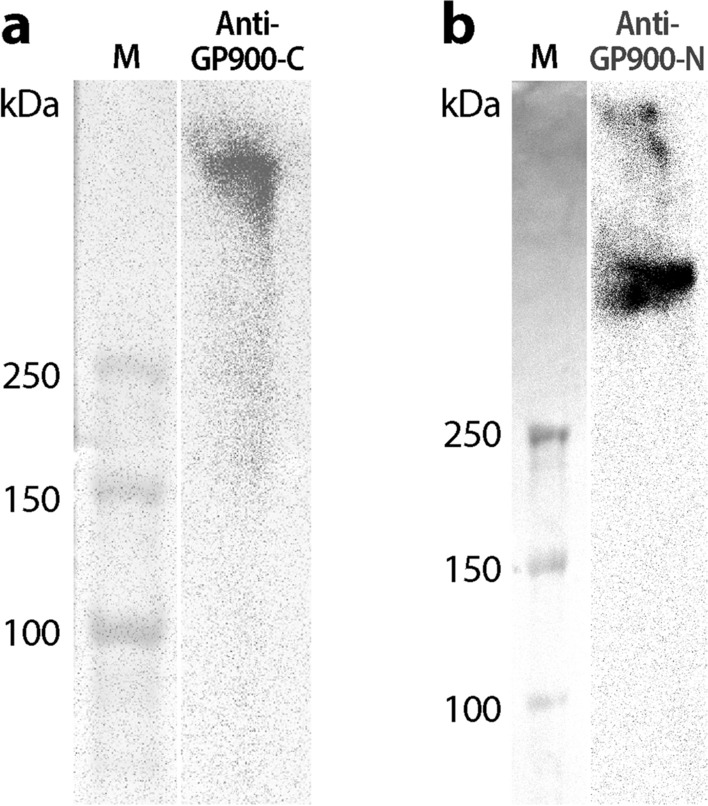
Western blot detection of native GP900 protein in the crude extracts of oocysts using affinity-purified anti-GP900-C polyclonal antibody (**a**) and anti-GP900-N monoclonal antibody (**b**). Note that the band recognized by each antibody was above the upper most band (250 kDa) in molecular markers (M)

GP900 is known to be “stored in micronemes prior to appearance on the surface of invasive forms” and discharged to form trails during the gliding locomotion of sporozoites [20, 21]. We were interested in confirming the relocation of GP900 from micronemes to the cell surface and testing whether this single-pass membrane protein was present on the sporozoite surface as a transmembrane protein in the cytoplasm membrane. Therefore, we raised two antibodies to distinguish the C-terminal cytoplasmic domain (rabbit anti-GP900-C pAb) from the N-terminal non-cytoplasmic domain (mouse anti-GP900-N mAb) (Fig. 1; dark red bars). Both anti-GP900-C pAb (affinity-purified) and anti-GP900-N mAb (prepared in the mouse ascites) recognized a single high-molecular-weight band above the 250-kDa marker in the western blot analysis (Fig. 3). The immunoblotting data were similar to those observed in earlier studies [1–3], confirming the specificity of the two antibodies.

**Fig. 4:**
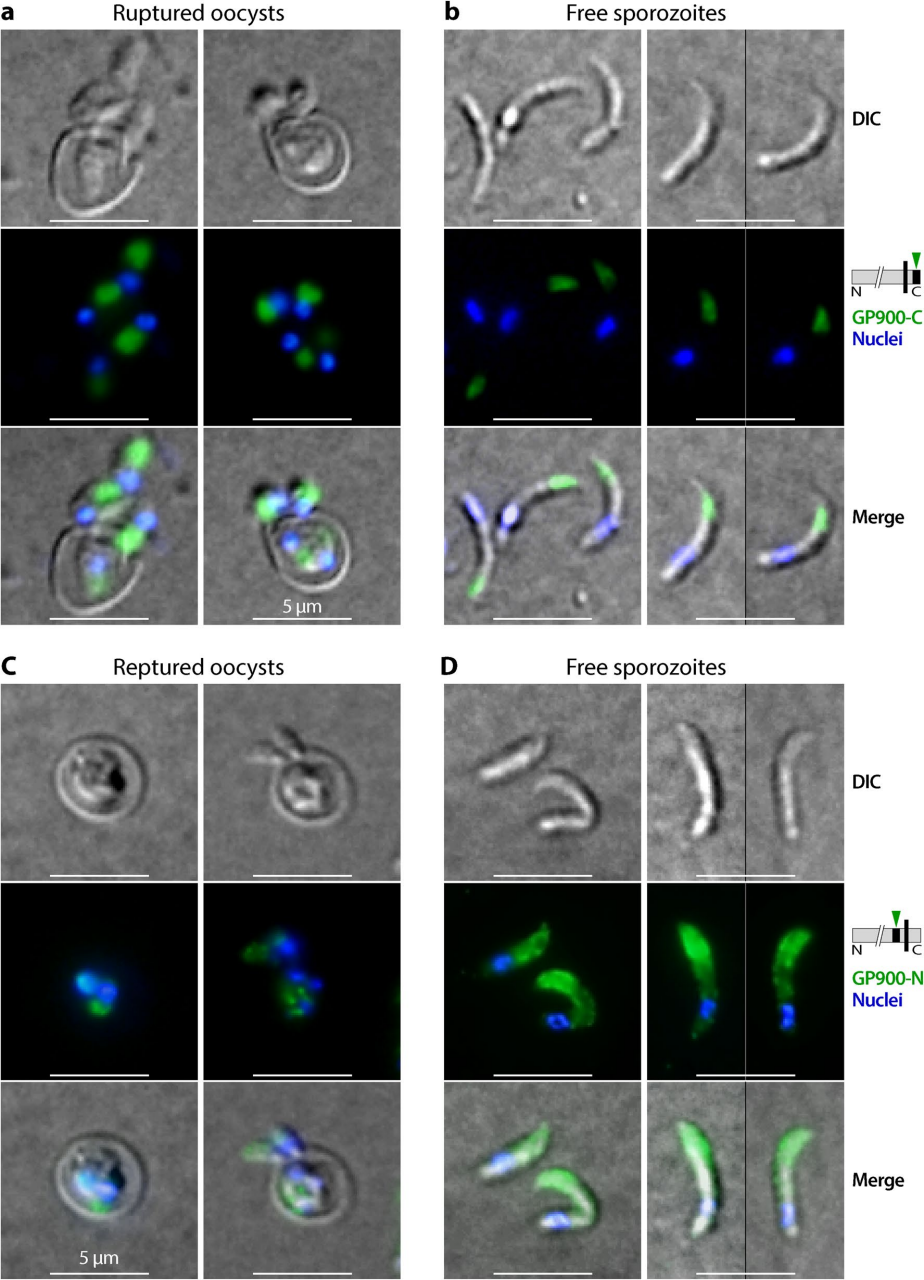
IFA detection of GP900 protein (green) in ruptured oocysts (**a**, **c**) and excysted sporozoites (**b**, **d**) using affinity-purified polyclonal anti-GP900-C (**a**, **b**) and monoclonal anti-GP900-N (**c**, **d**) antibodies. Nuclei (blue) were counterstained with DAPI. Ruptured oocysts were prepared by subjecting intact oocysts to three freeze–thaw cycles in 4% formaldehyde. Abbreviations: DAPI, 4,6-diamidino-2-phenylindole; DIC, differential interference contrast microscopy; IFA, immunofluorescence microscopy assay

**Fig. 5:**
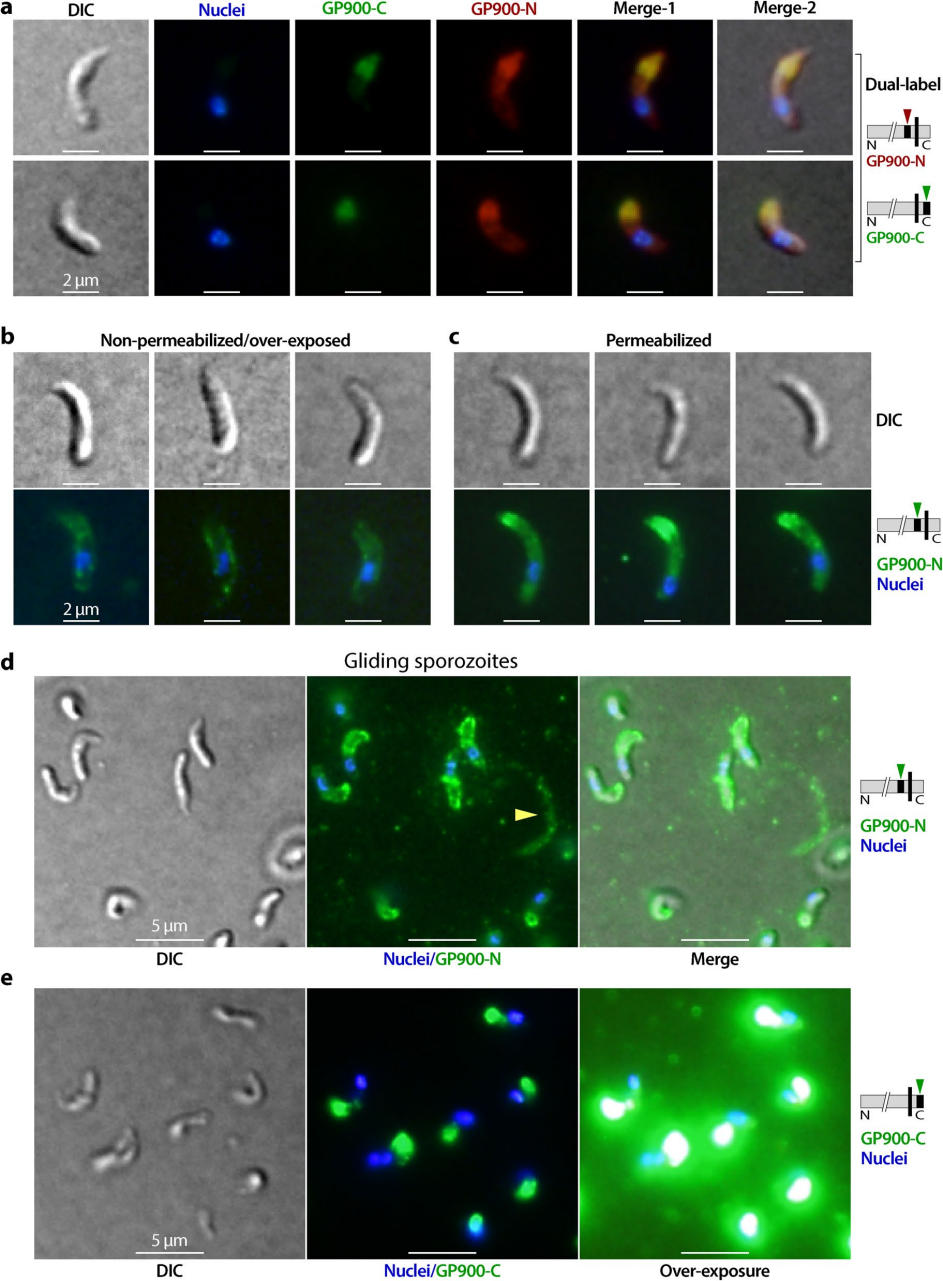
IFA detection of GP900 protein in excysted sporozoites. **a** Dual-labeling of GP900 with mouse anti-GP900-N (red) and rabbit anti-GP900-C (green) in excysted sporozoites. **b**, **c** IFA detection of GP900 protein on the external surface of sporozoites that had been fixed in formaldehyde but were unpermeabilized (**b**) in comparison with permeabilized sporozoites (**c**), using anti-GP900-N antibody. To facilitate a good comparison, both samples were processed in parallel under the same experimental conditions. It should be noted that weak, but clear when over-exposed, signals were produced in unpermeabilized sporozoites. **d**, **e** IFA detection of GP900 protein on the trails of gliding sporozoites using anti-GP900-N (**d**) and anti-GP900-C (**e**) antibodies; anti-GP900-N antibody stained the surface of sporozoites and some gliding trails (**d**) while anti-GP900-C antibody only stained the apical region of the gliding sporozoites (**e**). In all panels, nuclei were stained with DAPI

IFA using anti-GP900-C antibody confirmed the location of the protein in the apical region packed with micronemes in the sporozoites before and after excystation (Fig. 4a, b). In this assay, the immunolabeling of intact oocysts was initially obstructed by the impermeability of the oocyst walls, but ultimately resolved by rupturing the oocysts through treatment with freeze–thaw cycles to allow the access of antibodies to the sporozoites [9]. The anti-GP900-N antibody also chiefly labeled the apical region in sporozoites before excystation (as indicated by the presence on one side of the nuclei) (Fig. 4c). However, in excysted sporozoites, anti-GP900-N antibody not only strongly labeled the apical region, but also along the pellicle, with weaker but clear signals detected (Fig. 4d). Dual-labeling further confirmed the differential distributions of the two epitopes recognized by anti-GP900-C and anti-GP900-N antibodies in free sporozoites (Fig. 5a). The labeling of anti-GP900-N antibody on the surface of sporozoites was confirmed by IFA of formaldehyde-fixed, but non-permeabilized sporozoites (Fig. 5b), in which slightly weaker but clear fluorescent signals were observable on the surface of sporozoites, while no signals were detected inside of the sporozoites. For direct comparison, the fluorescent signals from permeabilized sporozoites were detected along the pellicles and inside the sporozoites that were processed in parallel under the same conditions (Fig. 5c). Furthermore, in the gliding sporozoites, anti-GP900-N antibody again labeled sporozoite pellicles and the gliding trails of some sporozoites (Fig. 5d), whereas anti-GP900-C antibody only labeled the apical region of sporozoites (Fig. 5e).

The IFA data can be summarized as follows. GP900 present in the micronemes of the sporozoite apical region contained both C-terminal cytoplasmic and N-terminal non-cytoplasmic domains (i.e. full-length GP900, minus the signal peptide in theory), whereas the GP900 protein distributed along the sporozoite surface or pellicles contained only the N-terminal non-cytoplasmic domain. The gliding sporozoites also discharged GP900 that lacked the C-terminal cytoplasmic domain. Based on these observations, we conclude that the C-terminal domain of GP900 is cleaved in the secretory pathway before it reaches the cell surface.

### GP900 is present in the asexual developmental stages, but absent in the sexual stages

**Fig. 6:**
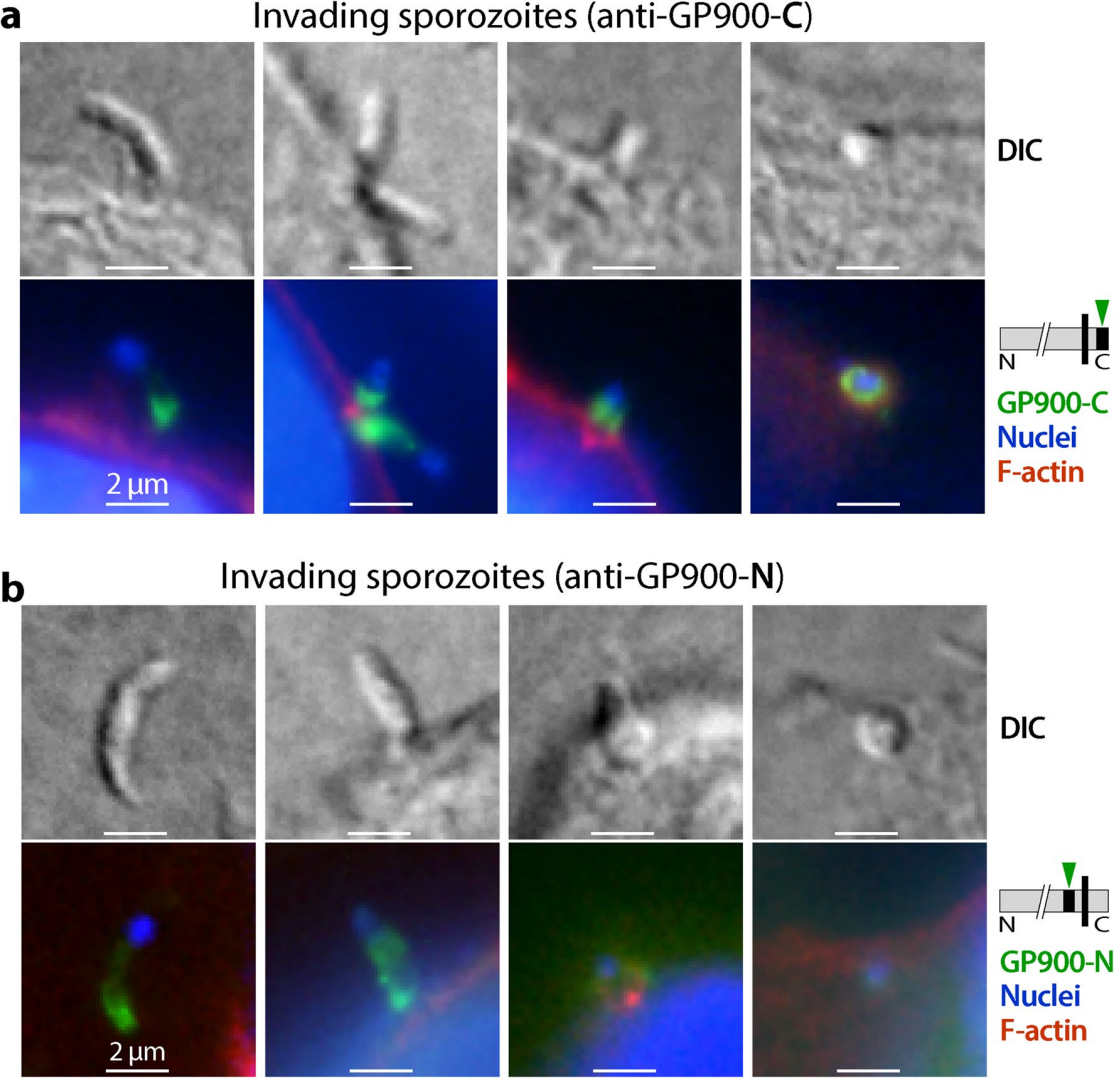
IFA detection of GP900 protein (green) in invading sporozoites using anti-GP900-C (**a**) and anti-GP900-N (**b**) antibodies. F-actin of host cells was stained with phalloidin–rhodamine (red), and nuclei were stained with DAPI (blue)

**Fig. 7:**
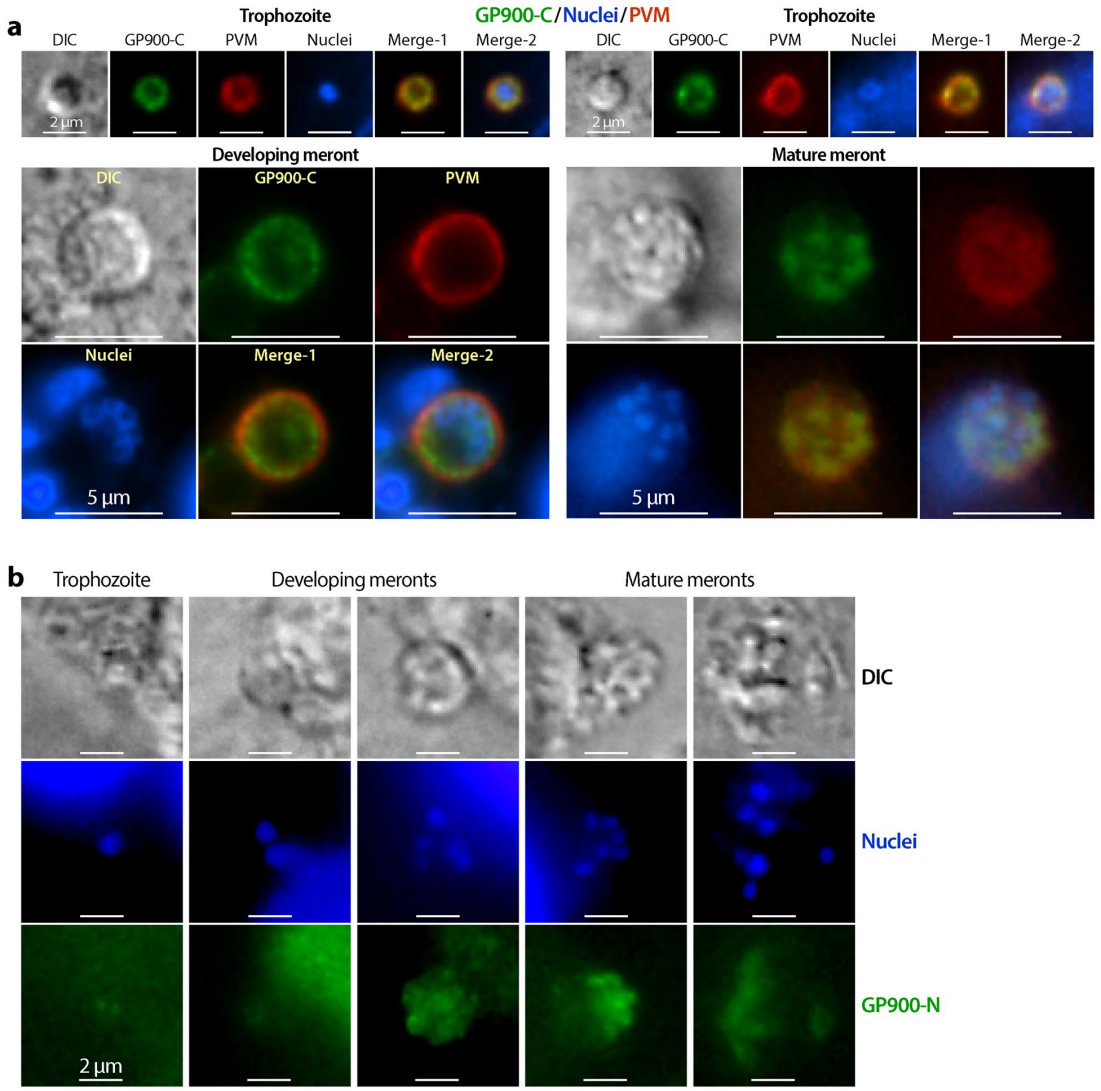
IFA detection of GP900 protein (green) in intracellularly developing parasites, including trophozoites and developing and mature meronts using anti-GP900-C (**a**) and anti-GP900-N (**b**) antibodies. **a** Parasites were co-labeled with a mouse clonal antibody against total parasite proteins, producing strong signals in the PVM (red). In both panels, nuclei were stained with DAPI. Abbreviations: PVM, Parasitophorous vacuole membrane

During the sporozoite invasion of host cells, anti-GP900-C antibody again labeled the apical region in the long sporozoites (early invasion), but became two dense spots in the short sporozoites (later invasion) or two semi-circles surrounding the newly formed trophozoites (Fig. 6a). Anti-GP900-N antibody also labeled the apical region (more strongly) plus the pellicles in the long sporozoites, but became more diffused in the short sporozoites and newly formed trophozoites (Fig. 6b). During *C. parvum* intracellular development, immunostaining by anti-GP900-C antibody was apparent (Fig. 7a, green), with signals detectable surrounding the nuclei in small meronts with one nucleus (Fig. 7a, upper two panels) or strong signals surrounding the large developing meronts with multiple nuclei (Fig. 7a, lower left panel). In mature meronts in which banana-like merozoites were formed, fluorescent signals became concentrated on one side of the nuclei, implying apical distributions in merozoites (Fig. 7a, lower right panel).

Co-staining of the PVM using a mouse polyclonal antibody against total *C. parvum* proteins (Fig. 7a, red) showed that the immunostaining by anti-GP900-C antibody was confined to the parasites within the PVM, rather than on the PVM. The IFA using anti-GP900-N antibody (Fig. 7b) revealed uneven signals in mature meronts, implying distributions on the apical region of merozoites, but weak signals in trophozoites and developing meronts. However, we also observed high background/non-specific signals from host cells using anti-GP900-N antibody that could not be resolved after multiple attempts of modifying the experimental conditions. In fact, anti-GP900-C antibody also produced relatively weak signals in developing meronts (vs stronger signals in developed meronts), but it produced low background/non-specific signals in host cells, allowing us to increase the exposure time to visualize the specific signals.

**Fig. 8:**
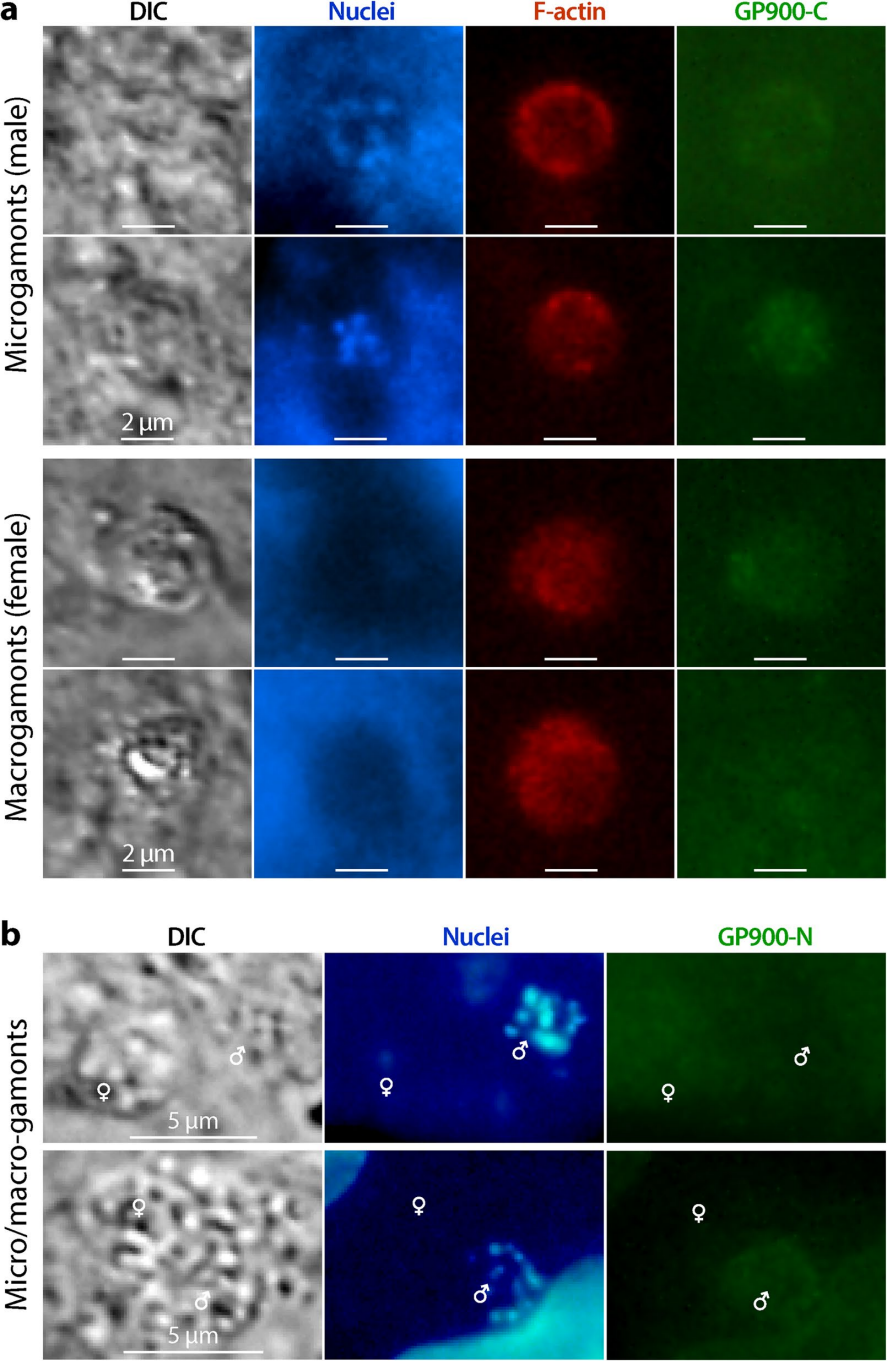
IFA detection of GP900 protein (green) in microgamonts and macrogamonts (sexual stages) using anti-GP900-C (**a**) and anti-GP900-N (**b**) antibodies. **a** Parasites were co-labeled with a mouse clonal antibody against total parasite proteins, producing strong signals in the PVM (red). In both panels, nuclei were stained with DAPI

While GP900 was observed in all asexual stages, including sporozoites and meronts, it was undetectable in the sexual stages of *C. parvum*. Neither anti-GP900-C nor anti-GP900-N antibodies produced signals in sexual stages, and both antibodies only produced close-to-the-background signals in macrogamonts and microgamonts (vs clear signals using the anti-total protein antibody) (Fig. 8).

### GP900 is released into the extracellular space by sporozoites and intracellular parasites

**Fig. 9:**
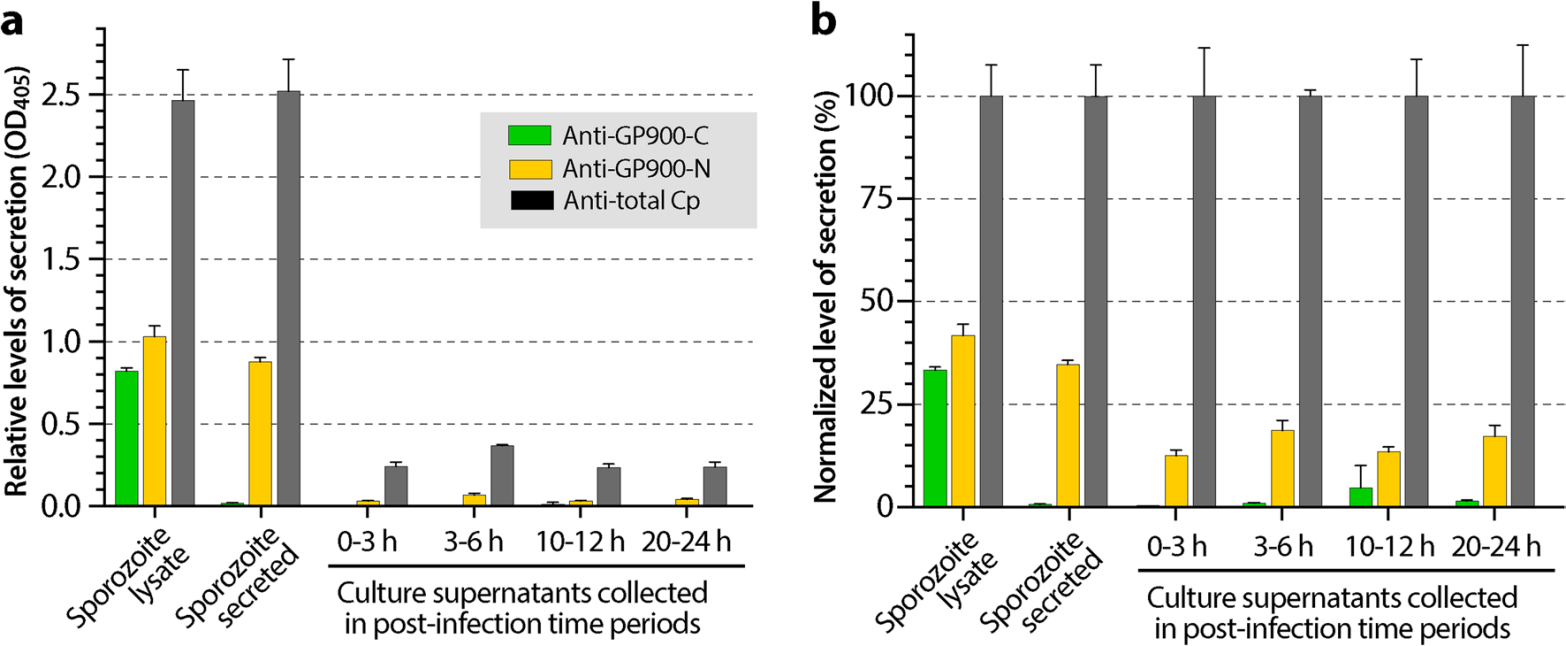
Detection of secreted GP900 protein from various parasite stages by ELISA. **a** Relative levels of secreted GP900 from excysted sporozoites (i.e. serum-free RPMI-1640 medium after incubation with free sporozoites following the removal of excystation medium) and intracellular parasites cultured for specified times (i.e. serum-free medium at between 0–3, 3–6, 10–12 and 20–24 hpi) using antibodies against GP900-C epitope (green), GP900-N epitope (orange) and total parasite proteins (gray). **b** The same optical density datasets from **a** after normalization with those derived from antibody against total parasite proteins. Abbreviations: ELISA, Enzyme-linked immunosorbent assay; hpi, hours post-infection

The lower signals from anti-GP900-N antibody in intracellular parasites were also implied based on the significant amount of processed/cleaved GP900 that was secreted/discharged into the extracellular space (i.e. into the culture medium), as determined by ELISA (Fig. 9). In this assay, anti-GP900-N antibody (Fig. 9, orange bars), but not anti-GP900-C antibody (green bars), detected secreted GP900 in the medium after incubation with excysted sporozoites (37 °C, 1 h) and in the serum-free medium collected from the invasion stage (i.e. 0–3 hpi) and from selected intracellular developmental stages (i.e. 0–3, 3–6, 10–12 and 20–24 hpi). Two positive reagent controls showed the expected results: (i) in the specimen control, both anti-GP900-N (orange bars) and anti-GP900-C (green bars) antibodies detected GP900 in the supernatants of sporozoite lysate prepared by the freeze–thaw treatment; and (ii) in the antibody control, the antibody raised against total parasite proteins detected antigens in all samples (gray bars). The OD values were relatively low for the intracellular samples (Fig. 9a), but still notable, particularly when individual datasets were normalized with the OD values obtained using anti-total protein antibody (Fig. 9b). Normalization with antiserum against total parasite proteins was less perfect, as the antiserum might not recognize all *C. parvum* proteins equally and different parasite stages might also secrete proteins unequally in specified time periods. However, this normalization provided a rough, but adequate comparison of the relative levels of secreted GP900 between various stages.

**Fig. 10:**
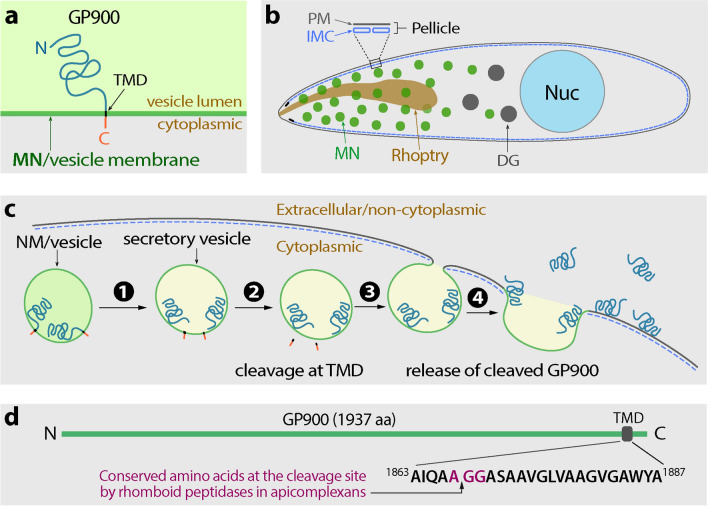
A working model for the biological process of GP900 in sporozoites. GP900 protein (**a**) is stored in the secretory organelle micronemes in sporozoites in oocysts before excystation (**b**). After excystation and during invasion, GP900 in the microneme vesicles enters the secretory pathway (**c**, step 1). Before the secretory vesicles reach to the plasma membrane, the short C-terminal cytoplasmic domain of GP900 is cleaved (step 2). The vesicles reach and fused with the plasma membrane (step 3), and cleaved GP900 molecules are discharged to the extracellular space (step 4). Some secreted GP900 molecules adhere to the surface of the plasma membrane due to the general adhesive property of the glycoprotein, while other molecules are shed into the surrounding microenvironment. The transmembrane domain of GP900 contains conserved amino acids (purple) near the non-cytoplasmic side for intramembrane cleavage by rhomboid peptidases (**d**). Abbreviations: DG, Dense granules; IMC, inner membrane complex; MN, microneme; Nuc, nucleus; PM, plasma membrane

Taking all of the ELISA and IFA data together, it is possible to build a working model for the biological process of GP900 in sporozoites (Fig. 10). First, in intact oocysts, this single-pass transmembrane glycoprotein is stored in the micronemes of sporozoites (Fig. 10a, b). Then, GP900 starts to enter the secretory pathway during/after excystation (Fig. 10c, step 1), in which the short C-terminal domain is cleaved at the TMD site (step 2) and the secretory vesicles cross the inner membrane complex consisting of flatted plates and fuses with the plasma membrane (step 3). Thirdly, the long N-terminal domain is released into the extracellular space (step 4). Because of the general adhesive property of a glycoprotein and the presence of charged amino acids, some discharged GP900 molecules are retained on the surface of the sporozoites. The cleavage is presumably carried out at the TMD by one of the three *C. parvum* rhomboid peptidases (CpROMs) (Fig. 10d), in which CpROM1 has been recently confirmed to be a micronemal protein as well [10]. In fact, GP900 contains an intramembrane sequence with a high identity to that of the known cleavage site of *Toxoplasma gondii* micronemal proteins (i.e. AA|GG in GP900 vs. IA|GG in TgMIC2 and TgMIC6) (Fig. 10d).

## Discussion

The mucin-like GP900 was discovered about 30 years ago as an immunodominant protein that was present in micronemes, but which relocated to the surface of sporozoites and subsequently shed from the surface of sporozoites during gliding locomotion [1–3]. However, little has been learned about the biology of GP900 during the past two decades. In this study, we were initially interested in determining how GP900 was shed from the surface of gliding sporozoites. We believed that this type I membrane protein was anchored to the sporozoite plasma membrane via the TMD, but cleaved from its surface based on the presence of gliding trails. Therefore, we raised two antibodies specific to the long N-terminal and short C-terminal domains, respectively, separated by the single TMD (i.e. anti-GP900-N and anti-GP900-C antibodies). Surprisingly, however, while the IFA revealed that both antibodies labeled the apical region of sporozoites, only anti-GP900-N antibody, but not anti-GP900-C antibody, labeled the sporozoite surface and gliding trails. This observation indicates that GP900 protein distributed along the sporozoite pellicles or surface lacks the C-terminal domain. Because both antibodies labeled the apical region of sporozoites in intact oocysts before excystation, the cleavage of the C-terminal domain should take place after GP900 enters the secretory pathway but before it reaches the surface of sporozoites during and after excystation.

Similar to classic mucins, GP900 is heavily glycosylated, but both *O*-glycans and *N*-glycans are relatively simple forms. For *O*-glycans, which is the major type of glycans in mucins and GP900, *C. parvum* lacks enzymes to further extend the *O*-GalNAc core, similar to the engineered “SimpleCell” lines that only express truncated *O*-GalNAc [5]. In the case of *N*-glycans, only a single arm is formed which is barely processed in the ER or Golgi body [6]. Therefore, GP900 contains a much lower amount of polysaccharides than classic mucins, making it less expandable in the water-containing extracellular environment. The infection sites of *C. parvum* are human or animal intestinal epithelial cells covered by a thick layer of mucus consisting mainly of mucin-2 that is negatively charged due to the presence of sialic acid [22, 23]. Therefore, excysted sporozoites need to interact with host mucins during the former’s migration through the mucus layer to reach the surface of the host cell. Because *O*-glycans are unextended, GP900 lacks the terminal negatively charged sialic acid that is found in intestinal mucins, although it may be slightly negatively charged based on the theoretical isoelectric point (pI: 4.62). This physiochemical property is favorable for the diffusion of released GP900 molecules at the infection sites by avoiding electrostatic repulsion from the highly negatively charged intestinal mucins. In the gliding trail IFA assay, the diffusion of GP900 molecules into the medium also explains why the trails were detected only from some (but not all) gliding sporozoites (Fig. 5d).

In previous studies, GP900 was reported to be one of the glycoproteins that tether sporozoites to the inner surface of the oocyst wall, based on the results of IFA and IEM assays [4]. However, in the present study, both anti-GP900-C and anti-GP900-N antibodies failed to label the inner side of or around the oocyst walls in ruptured oocysts (Fig. 4). We noted that Chatterjee and colleagues used two monoclonal antibodies in their study that were specific to an *N*-linked epitope (mAb 4G12) and *O*-linked GalNAc (mAb 4E9), rather than specific to GP900 only [4]. Therefore, the antigens recognized by 4G12 and 4E9 antibodies could be other glycoprotein containing *N*- or *O*-glycans, rather than GP900. In other words, glycoproteins are present, but not GP900, that tether sporozoites and oocyst walls. This notion is also supported by the absence of adhesive domains in GP900 necessary for binding/tethering molecules (Fig. 1), such as the APPLE, TSP, SUSHI and EGF domains that are commonly present in adhesion proteins [24, 25].

Based on the physiological properties (mucin-like, weakly charged and weakly adhesive), the presence in sporozoites and developed merozoites, and the discharge of cleaved molecules to the surface of sporozoites and extracellular space, we speculate that GP900 mainly plays a lubrication role during the gliding locomotion, invasion and intracellular development.

## Conclusions

Using IFA with two antibodies to the long N-terminal non-cytoplasmic domain (anti-GP900-N) and the short C-terminal cytoplasmic domain (anti-GP900-C), we have confirmed that the mucin-like glycoprotein GP900 in *C. parvum* is stored in the micronemes of sporozoites and merozoites. We have also noted that its short cytoplasmic domain is cleaved in the secretory pathway before the protein is released to the extracellular space of sporozoites during/after excystation. The molecular properties and the behavior of GP900 in different developmental stages also imply that this protein mainly plays a lubrication role.

## Abbreviations

CpGP900: Cryptosporidium parvum glycoprotein GP900
GR: Dense granules
ER: Endoplasmic reticulum
GalNAc: N-Acetylgalactosamine
GP900-C: Cryptosporidium parvum glycoprotein GP900 C-terminus
GP900-N: Cryptosporidium parvum glycoprotein GP900 N-terminus
HBC: Hyperimmune bovine colostral
IFA: Immunofluorescence assay
IEM: Immuno-electron microscopy
IMC: Inner membrane complex
mAb: Monoclonal antibody
MN: Microneme
pAb: Polyclonal antibody
PM: Plasma membrane
PV: Parasitophorous vacuole
PVM: Parasitophorous vacuole membrane
SP: Signal peptide
TMD: Transmembrane domain
